# Efficacy of SGLT2 inhibitors in glycemic control, weight loss and blood pressure reduction: a systematic review and meta-analysis

**DOI:** 10.1186/1758-5996-7-S1-A58

**Published:** 2015-11-11

**Authors:** Lana Catani Pinto, Dimitris Varvaki Rados, Luciana Reck Remonti, Caroline Kaercher Kramer, Cristiane Bauermann Leitao, Jorge Luiz Gross

**Affiliations:** 1 Hospital de Clínicas de Porto Alegre, Porto Alegre, Brazil

## Background

Sodium–glucose cotransporter 2 inhibitors (SGLT2i) are a novel antidiabetic class that inhibits glucose reabsorption and produce glycosuria. These medications are being increasingly used as dual therapy with metformin for type 2 diabetes (T2D) treatment, due to their beneficial effect on weight and blood pressure. Three agents are approved for clinical use and they may differ on potency due to inhibition of only renal or both renal and bowel glucose transportation.

## Objective

to evaluate the efficacy of SGLT2i, dapagliflozin (DAPA), canagliflozin (CANA) and empagliflozin (EMPA), on HbA1c, weight and blood pressure (BP) in comparison with placebo and other antidiabetic medications.

## Materials and methods

MEDLINE, Cochrane central and EMBASE databases were searched for randomized clinical trials (RCTs) including patients with T2D allocated to SGLT2i for at least 12 weeks. A direct and network meta-analysis (NMA) were conducted.

## Results

**Figure 1:**
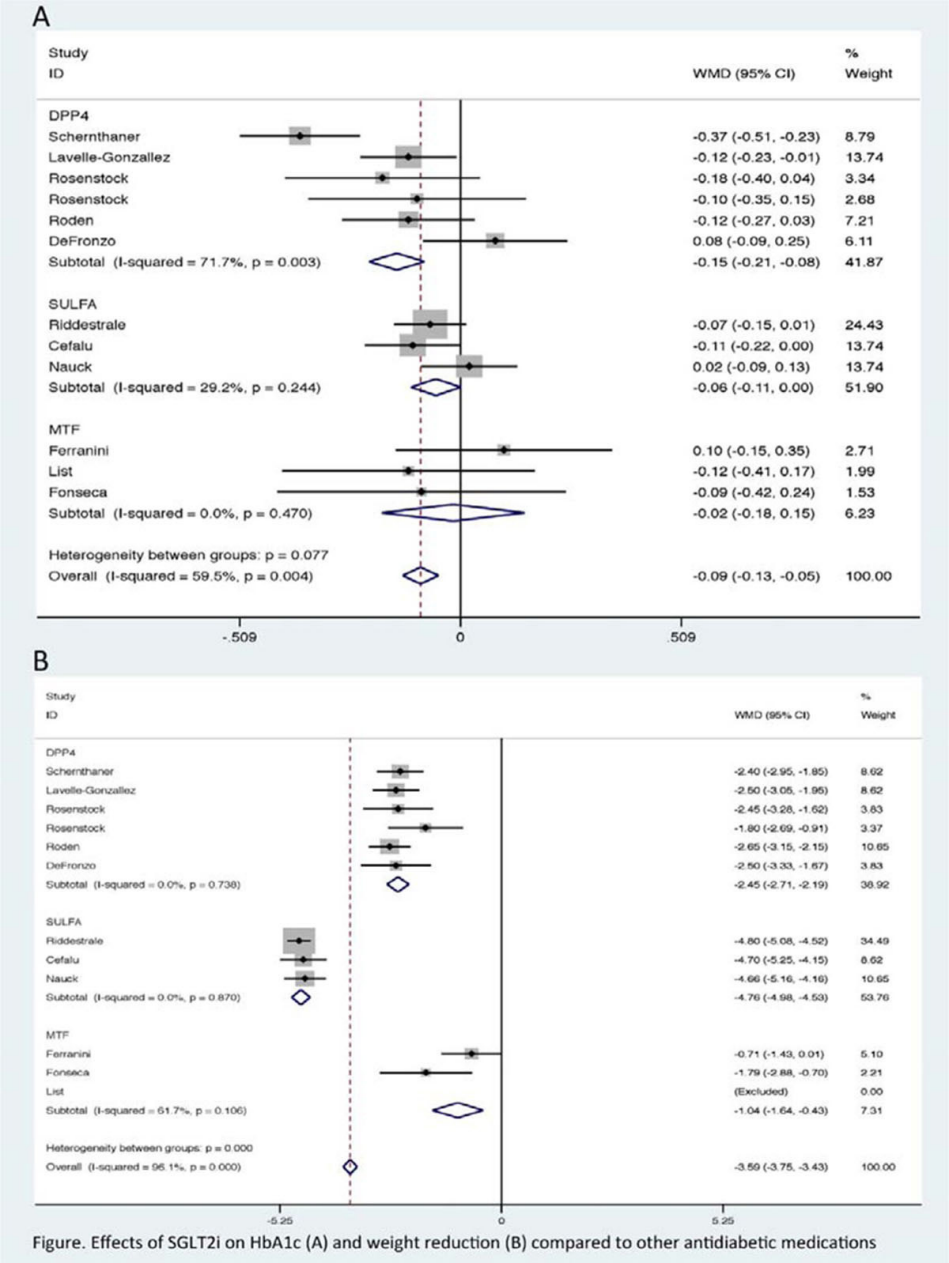
Effects of SGLT2i on HbA1c (A) and weight reducAon (B) compared to other antidiabetic medications

Thirty-nine RCTs were included (25.468 patients). CANA 300 mg, EMPA 25 mg and DAPA 10 mg were associated with better g.lycemic control (HbA1c -1.01%, -0.69%, -0.51%, respectively), and weight loss (-2.66 kg; -1.81 kg; -1.80 kg, respectively) when compared to placebo. In NMA, CANA 300 mg was superior to the others SGLT2i for HbA1c (EMPA 25 mg: - 0.22% and DAPA 10 mg: -0.26%), and weight (EMPA 25 mg: -1.06 kg and DAPA 10 mg: -0.84 kg). CANA 300 mg and DAPA 10 mg decreased systolic BP (-4.77 mmHg and -2.66 mmHg, respectively) and diastolic BP (-1.99 mmHg and -1.76 mmHg, respectively) in comparison to placebo. SGLT2i were similar to metformin and sulphonylurea regarding to HbA1c, but superior to DPP4 inhibitors (-0.15%) (Figure 1). Furthermore, SGLT2i were better than metformin, sulphonylurea, and DPP4 inhibitors for reduction of weight (-1.04 kg, -4.76 kg and - 2.45 kg, respectively) and systolic BP (-5.86 mmHg, -5.44 mmHg e -4.43 mmHg, respectively). SGLT2i were also better than sulphonylurea and DPP4 inhibitors for diastolic BP lowering (-2.59 mmHg, -1.89 mmHg, respectively). Initial combination of SGLT2i plus metformin resulted in greater reduction of HbA1c than SGLT2 plus DPP4i (-0.53% vs. -0.19%).

## Conclusion

In T2D patients, SGLT2i were superior to placebo for all outcomes analyzed, and CANA seems to be the most potent among then. SGLT2i are as effective as metformin and sulphonylurea and superior to DPP4 inhibitors for HbA1c, but more potent than these classes regarding weight and BP reduction.

